# Understanding the role of cerebellum in early Parkinson’s disease: a structural and functional MRI study

**DOI:** 10.1038/s41531-024-00727-w

**Published:** 2024-06-19

**Authors:** S. Pietracupa, A. Ojha, D. Belvisi, C. Piervincenzi, S. Tommasin, N. Petsas, M. I. De Bartolo, M. Costanzo, A. Fabbrini, A. Conte, A. Berardelli, P. Pantano

**Affiliations:** 1 IRCCS Neuromed, Pozzilli, IS Italy; 2 Department of Human Neuroscience, Sapienza University of Rome, Rome, Italy; 3 Department of Public Health and Infectious Disease, Sapienza University of Rome, Rome, Italy

**Keywords:** Parkinson's disease, Parkinson's disease

## Abstract

Increasing evidence suggests that the cerebellum may have a role in the pathophysiology of Parkinson’s disease (PD). Hence, the scope of this study was to investigate whether there are structural and functional alterations of the cerebellum and whether they correlate with motor and non-motor symptoms in early PD patients. Seventy-six patients with early PD and thirty-one age and sex-matched healthy subjects (HS) were enrolled and underwent a 3 T magnetic resonance imaging (MRI) protocol. The following MRI analyses were performed: (1) volumes of 5 cerebellar regions of interest (sensorimotor and cognitive cerebellum, dentate, interposed, and fastigial nuclei); (2) microstructural integrity of the cerebellar white matter connections (inferior, middle, and superior cerebellar peduncles); (3) functional connectivity at rest of the 5 regions of interest already described in point 1 with the rest of brain. Compared to controls, early PD patients showed a significant decrease in gray matter volume of the dentate, interposed and fastigial nuclei, bilaterally. They also showed abnormal, bilateral white matter microstructural integrity in all 3 cerebellar peduncles. Functional connectivity of the 5 cerebellar regions of interest with several areas in the midbrain, basal ganglia and cerebral cortex was altered. Finally, there was a positive correlation between abnormal functional connectivity of the fastigial nucleus with the volume of the nucleus itself and a negative correlation with axial symptoms severity. Our results showed that structural and functional alterations of the cerebellum are present in PD patients and these changes contribute to the pathophysiology of PD in the early phase.

## Introduction

Parkinson’s disease (PD) is a neurological condition due to the degeneration of dopaminergic neurons of the substantia nigra pars compacta, causing a basal ganglia-thalamo-cortical loop dysfunction^1–4^.

However, in the last decade, there has been increasing evidence suggesting that also the cerebellum is involved in the pathophysiology of PD^5–7^. The cerebellum may play a role in the pathophysiology of motor signs (including motor execution and motor learning), gait disturbances, dyskinesias and non-motor signs (cognitive disturbances)^6,8^. Also, tremor has been linked to abnormal functional connectivity between the basal ganglia and the cerebellum via the thalamus and the cerebral cortex^9,10^. More importantly, recent studies have also shown that the cerebellum is anatomically and reciprocally connected with the basal ganglia^8,11,12^.

Most previous studies on PD investigated the cerebellar function without examinations of specific nuclei and lobules. It is therefore unclear whether distinct cerebellar regions are specifically involved in PD and whether they contribute to the pathophysiology of motor and non-motor symptoms. One further issue that is still unclear is whether possible changes in cerebellar structures and functions are directly implicated in the pathophysiology of the disease or whether the involvement of the cerebellum reflects a compensatory activity to limit or delay the progression of the motor disturbances of PD^10^. One way to address this latter point is to investigate whether cerebellar changes are already present in the early stages of the disease.

To better understand the cerebellum’s role in the pathophysiology of PD, we performed a magnetic resonance investigation of the cerebellum in a sample of early PD patients. We investigated the structural and functional characteristics of five specific cerebellar regions (sensorimotor cerebellum, cognitive cerebellum, dentate, fastigial, and interposed nuclei) and the structural integrity of cerebellar fiber connections passing through the superior, middle and inferior cerebellar peduncles (SCP, MCP, ICP). Lastly, we investigated possible correlations between neuroimaging findings and motor and non-motor symptoms. The results were compared to those of a group of healthy subjects.

## Results

There were no significant differences in age (*p* = 0.08) or sex (*p* = 0.56) between early PD patients and HS. In patients, the mean MDS-UPDRS score was 17.17 ± 8.52. UPDRS subscore values, as well as mean NMSS, MoCA, FAB and BDI scores are provided in Table 1. Accordingly, in the patient sample obtained from PPMI dataset there were no differences in age (*p* = 0.87) or sex (*p* = 0.26) between PD de novo patients and HS at baseline as well as between our PD de novo patient sample and PPMI subjects (age: *p* = 0.91; sex: *p* = 0.26). Clinical and demographic data of patients obtained from PPMI dataset are described in Supplementary Table 9.

**Table 1:** Demographic and clinical characteristics of healthy subjects (HS) and early PD patients

Demographic /clinical feature	HS (mean ± SD)	Early PD (mean ± SD)	p-value
Age (years)	64.23 ± 9.32	60.6 ± 9.68	0.08
Sex (F/M)	12/19	23/53	0.56
Disease duration (years)	–	0.95 ± 1.05	–
Age at onset	–	59.73 ± 9.07	–
UPDRS III	–	17.17 ± 8.52	–
Bradykinesia [median (range)]	–	6 (0–20)	–
Tremor [median (range)]	–	2 (0–6)	–
Posture [median (range)]	–	0 (0-3)	–
Gait [median (range)]	–	1 (0–2)	–
MOCA	–	27.33 ± 2.44	–
FAB	–	16.5 ± 3.22	–
BDI	–	4.03 ± 4.81	–
NMSS	–	30.44 ± 30.14	–
H&Y	–	1.33 ± 0.56	–

*MDS-UPDRS-III* Movement Disorder Society-sponsored revision of the Unified Parkinson’s Disease Rating Scale, part III, *MoCA* Montreal Cognitive Assessment, *FAB* Frontal Assessment Battery, *BDI* Beck Depression Inventory, *NMSS* Non-Motor Symptoms Scale (NMSS), *H&Y* Hoehn and Yahr Scale.

### Cerebellar volumes

**Fig. 1:**
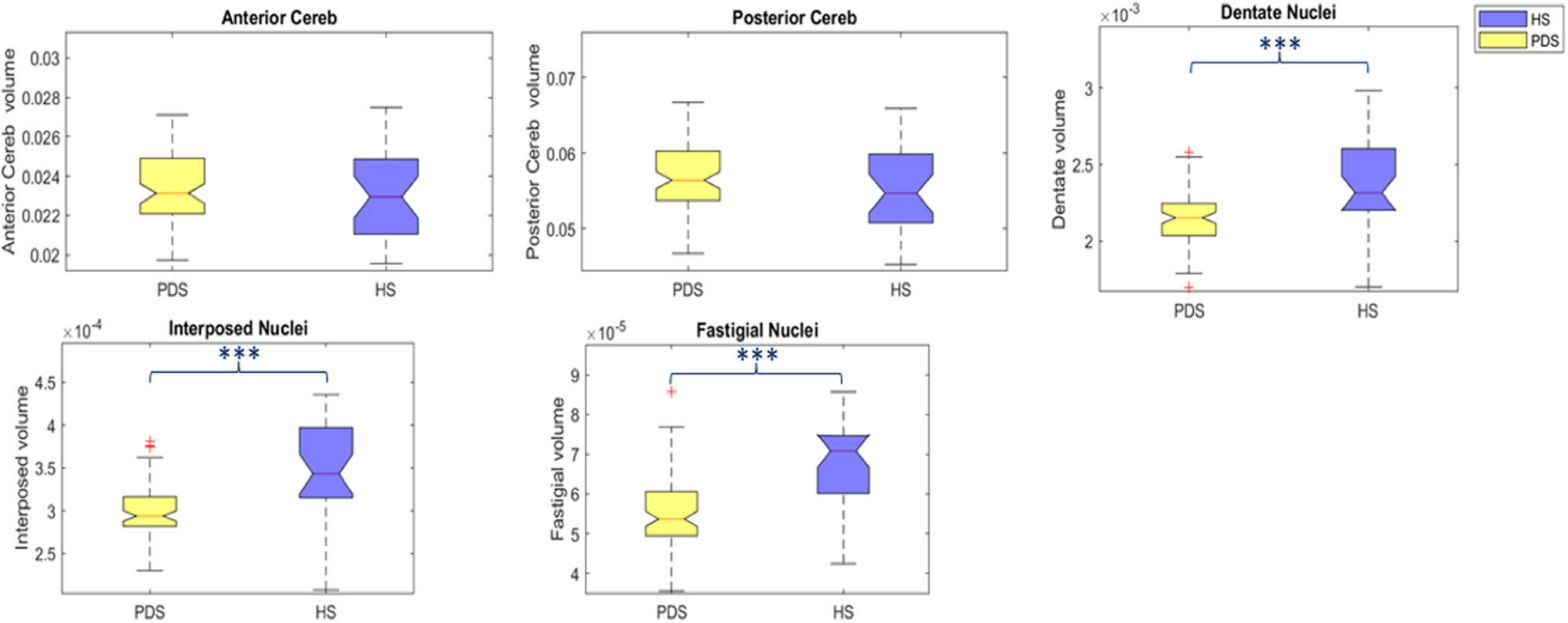
Cerebellar lobular analysis. Boxplots depict the cerebellar volumetric differences between early PD patients and HS. The early PD group was indicated by the yellow color and the HS one was indicated by the blue color at (**a**) sensory-motor cerebellum, (**b**) cognitive cerebellum, (**c**) dentate nuclei, (**d**) interposed nuclei, (**e**) fastigial nuclei. The median marks the midpoint of the data, and it is shown by the red line. The upper and lower whiskers represent the 75th and 25th percentiles, respectively. The whiskers extend to the most extreme data points not considered outliers, and the outliers are plotted individually using the “+” symbol.

PD patients had significantly lower dentate, fastigial, and interposed nuclei volumes than HS. Conversely, lobular volumes of the sensorimotor and cognitive cerebellum did not significantly differ from those of HS. (Fig. 1, Table 2).

**Table 2:** Cerebellar lobules and nuclei volumes in early PD and healthy control (HS)

	Early PD (mean ± SD)	HS (mean ± SD)	*p*-value
Sensory-motor cerebellum	0.0234 ± 0.0018	0.0229 ± 0.0022	0.17
Cognitive cerebellum	0.0567 ± 0.0042	0.0549 ± 0.0057	0.09
Dentate nucleus	0.0021 ± 0.0001	0.0023 ± 0.0002	**<0.001**
Interposed nucleus	0.0003 ± 3.1e−05	0.0003 ± 5.4e−05	**<0.001**
Fastigial nucleus	5.5e−05 ± 9e−06	6.7e-05 ± 1.1e−05	**<0.001**

Differences between groups were assessed using the Mann–Whitney *U* test, FDR corrected for multiple comparisons. Raw sensory-motor cerebellum, cognitive cerebellum, bilateral dentate, interposed and fastigial nuclei volumes were normalized within each subject as a ratio of intracranial volume and reported as a fraction. FDR-corrected *p* values as <0.05 is in bold.

### Diffusion tensor Imaging of cerebellar peduncles

**Fig. 2:**
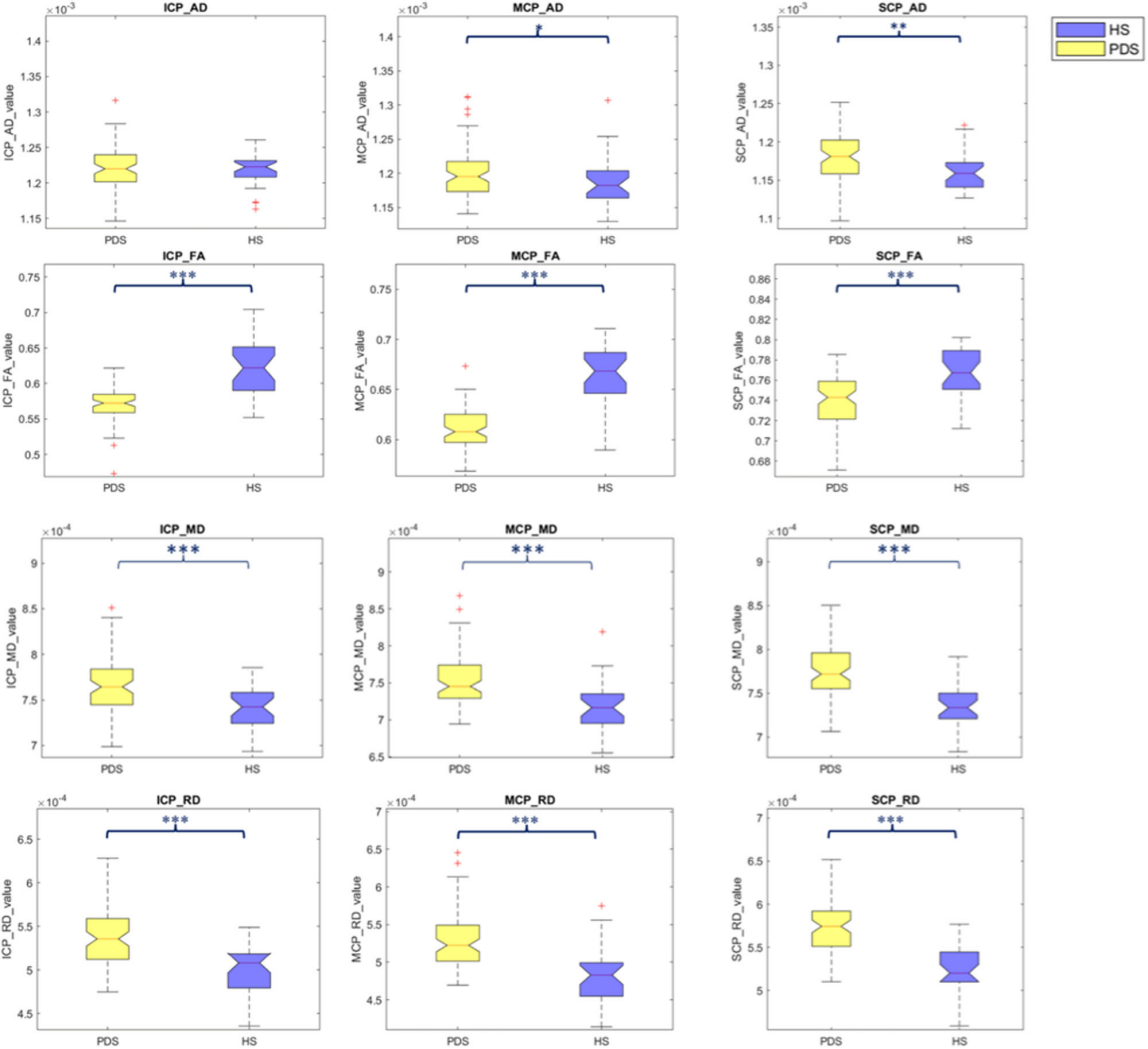
Cerebellar peduncles white matter analysis. The figure depicts axial diffusivity (AD), fractional anisotropy (FA), mean diffusivity (MD), and radial diffusivity (RD) alterations between early PD patients and HS within three cerebellar tracts: inferior cerebellar peduncle (ICP), middle cerebellar peduncle (MCP), and superior cerebellar peduncle (SCP). The early PD group was indicated by the yellow color and the HS one was indicated by the blue color. The median marks the data’s midpoint and is shown by the red line. The upper and lower whiskers represent the 75th and 25th percentiles, respectively. The whiskers extend to the most extreme data points not considered outliers, and the outliers are plotted individually using the “+” symbol.

Compared to HS, early PD patients exhibited lower FA and higher MD and RD values in all three cerebellar peduncles. Early PD patients also exhibited higher AD values in MCP and SCP (Fig. 2, Table 3).

**Table 3:** DTI measures (AD, FA, MD, RD) of the WM tracts of the middle, superior, and inferior cerebellar peduncles in HS and early PD patients

	Early PD (mean ± SD)	HS (mean ± SD)	*p* value
*AD*			
ICP	0.00122 ± 0.00003	0.00122 ± 0.00002	0.9721
MCP	0.00120 ± 0.00003	0.00119 ± 0.00004	**0.0282**
SCP	0.00118 ± 0.00003	0.00116 ± 0.00003	**0.0035**
*FA*			
ICP	0.57039 ± 0.02536	0.62197 ± 0.03794	**<0.001**
MCP	0.61020 ± 0.01920	0.66239 ± 0.03254	**<0.001**
SCP	0.73918 ± 0.02747	0.76898 ± 0.02443	**<0.001**
*MD*			
ICP	0.00076 ± 0.00003	0.00074 ± 0.00003	**<0.001**
MCP	0.00075 ± 0.00003	0.00078 ± 0.00004	**<0.001**
SCP	0.00078 ± 0.00003	0.00074 ± 0.00003	**<0.001**
*RD*			
ICP	0.00054 ± 0.00003	0.00050 ± 0.00003	**<0.001**
MCP	0.00053 ± 0.00003	0.00048 ± 0.00004	**<0.001**
SCP	0.00057 ± 0.00003	0.00053 ± 0.00003	**<0.001**

Differences between groups were assessed using the Mann–Whitney *U* test, FDR corrected for multiple comparisons. FDR-corrected *p* values as <0.05 is in bold.

*AD* axial diffusivity, *FA* fractional anisotropy, *MD* mean diffusivity, *RD* radial diffusivity, *SCP* superior cerebellar peduncle, *MCP* middle cerebellar peduncle, *ICP* inferior cerebellar peduncle.

### Cerebellar functional connectivity

#### Sensorimotor cerebellum

**Fig. 3:**
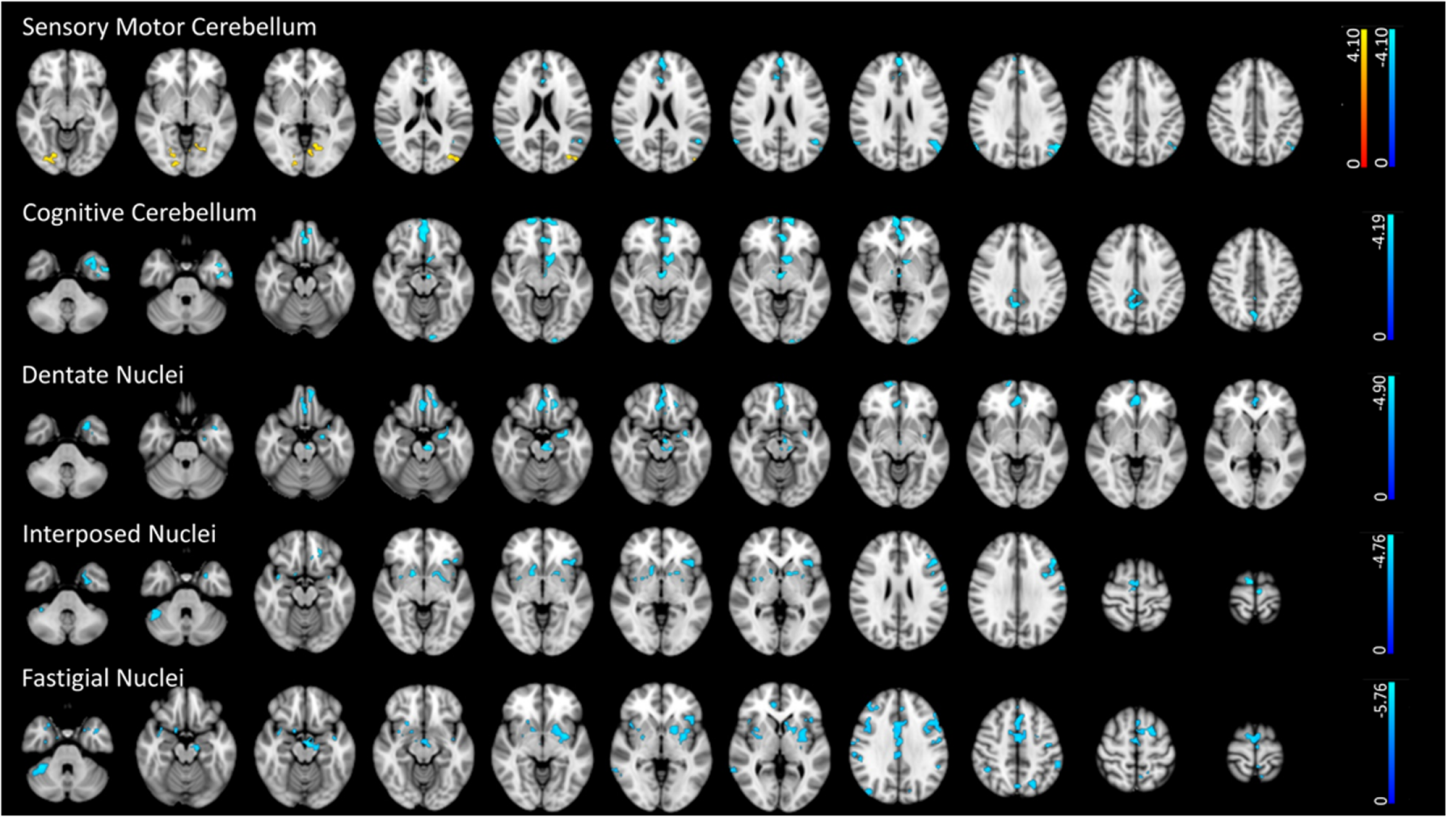
Cerebellar functional connectivity differences between early PD patients and HS. The figure depicts group differences (*p* < 0.01, FDR corrected) in functional connectivity (FC) between early PD patients and HS in the selected 5 ROIs: (**a**) Sensory-motor cerebellum, (**b**) Cognitive cerebellum, (**c**) Dentate Nucleus, (**d**) Interposed nucleus, (**e**) Fastigial Nucleus. Light blue indicates areas of reduced FC (in early PD patients), while red indicates areas of increased FC (in early PD patients) superimposed on the MNI152 standard brain. The color bars represent *t* values.

PD patients showed significantly higher FC with the occipital cortex bilaterally, right fusiform gyrus and bilateral lingual gyrus (Fig. 3a, Supplementary Table 1) than HS. The sensorimotor cerebellum also showed lower FC with bilateral angular and superior frontal gyri and bilateral cingulate and left paracingulate gyrus when compared to HS (Fig. 3a, Supplementary Table 2).

#### Cognitive cerebellum

PD patients showed significantly lower FC with cortical (frontal cortex, precuneus, temporal gyrus, occipital pole) and subcortical areas (accumbens, thalamus and midbrain) (Fig. 3b, Supplementary Table 3) than HS.

#### Dentate nuclei

PD patients showed significantly lower FC with cortical (frontal and temporal cortices, and cingulate and paracingulate gyrus) and subcortical areas (midbrain and amygdala) (Fig. 3c, Supplementary Table 4) in comparison to HS.

#### Interposed nuclei

PD patients showed significantly lower FC with cortical (supplementary motor cortex, insula, superior frontal gyrus, pre- and post-central gyrus) and subcortical areas (putamen, caudate and pallidum, and right cerebellar Crus I) (Fig. 3d, Supplementary Table 5) than HS.

#### Fastigial nuclei

PD patients showed significantly lower FC with cortical (supplementary motor cortex, frontal gyri, pre-central gyrus, cingular and insular cortices, and precuneus) and subcortical areas (midbrain, putamen, amygdala, caudate and pallidum, and right cerebellar Crus I) compared to HS (Fig. 3e, Supplementary Table 6) than HS.

### Correlation analyses

No significant correlations were found between cerebellar volumes and WM damage of the three cerebellar peduncles, neither between cerebellar FC abnormalities and WM damage of the three cerebellar peduncles.

**Fig. 4:**
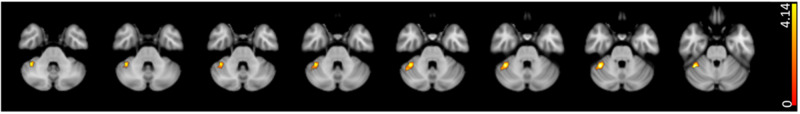
Correlation of altered fastigial FC with fastigial GM volume. The figure shows the positive correlation (red-yellow color code, *p* < 0.01, FDR corrected) between fastigial FC and fastigial GM volume.

**Fig. 5:**
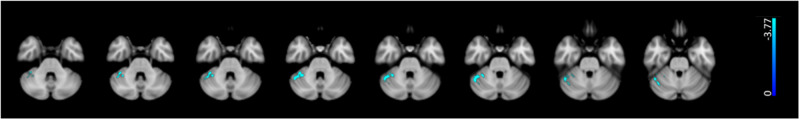
Correlation of fastigial FC with posture score. The figure shows the negative correlation (blue-light-blue color code, *p* < 0.01, FDR corrected) of fastigial FC with posture score in early PD patients.

On the other hand, the FC of the fastigial nucleus with right cerebellar lobule VI and crus I positively correlated with fastigial GM volume (Fig. 4, Supplementary Table 7). Also, the FC of the fastigial nucleus with right cerebellar lobule VI and crus I negatively correlated with posture score (Fig. 5, Supplementary Table 8). Altered FC of the other ROIs did not significantly correlate with any of the investigated clinical scores. Lastly, no correlations were also found between cerebellar volumes/WM damage and clinical scores.

### Supplementary functional connectivity analysis

**Fig. 6:**
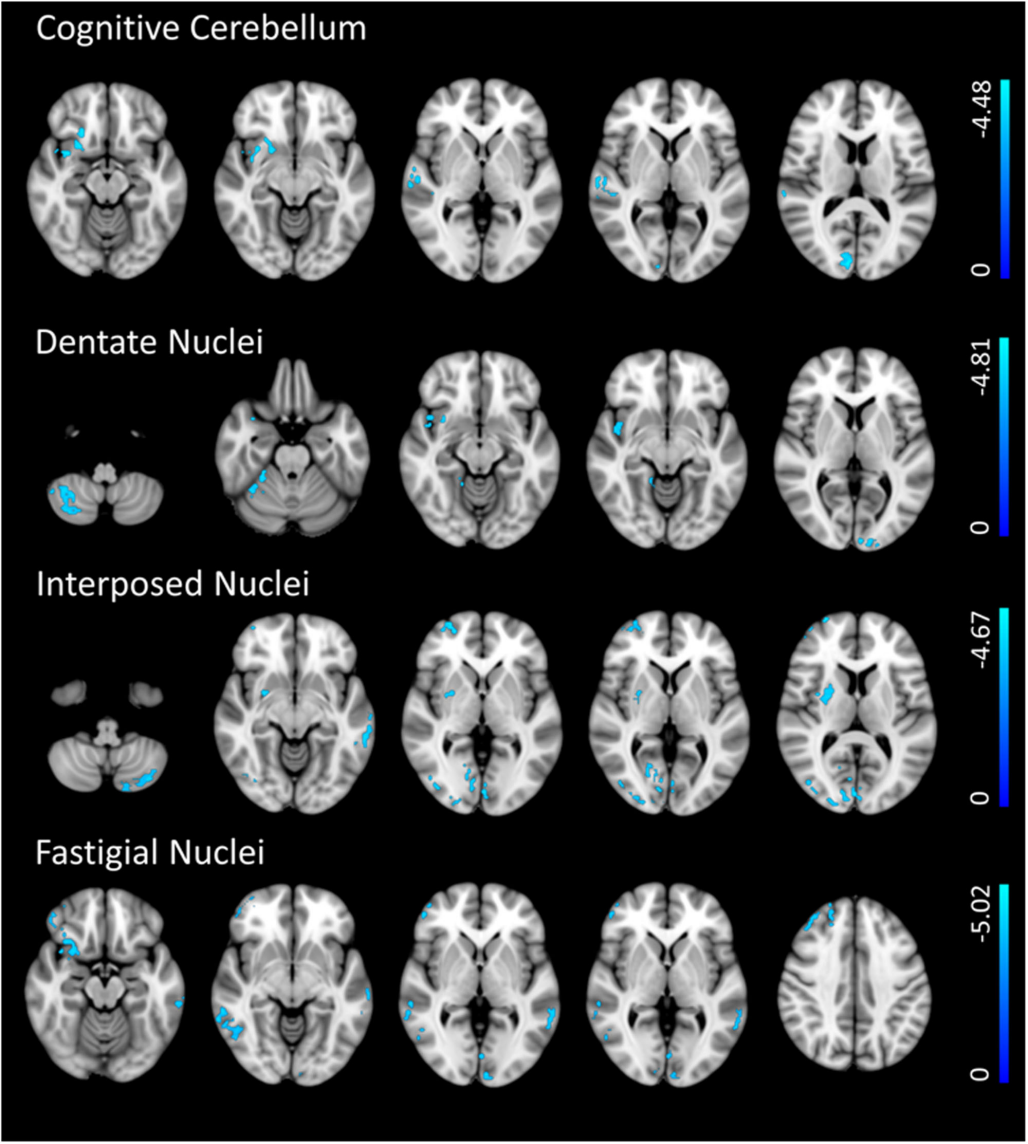
Cerebellar functional connectivity differences between early PD patients and HS (PPMI dataset). The figure depicts group differences (*p* < 0.01, FDR corrected) in functional connectivity (FC) between early PD patients and HS in the selected 5 ROIs: (**a**) Cognitive cerebellum, (**b**) Dentate Nucleus, (**c**) Interposed nucleus, (**d**) Fastigial Nucleus. Light blue indicates areas of reduced FC (in early PD patients).

Results obtained from the supplementary analysis on PPMI data confirmed a decreased connectivity between cerebellar ROIs and several brain regions at baseline. No areas of increased cerebellar FC were observed. Results are shown in Supplementary Tables 10–13 and Fig. 6.

## Discussion

Overall, our results indicate the presence of structural and functional changes in the cerebellum in PD patients in their early stages. We found GM volume reduction of bilateral dentate, fastigial, and interposed nuclei and microstructural changes in the three cerebellar peduncles. Furthermore, we found altered functional connectivity of the sensorimotor and cognitive cerebellum as well as the three nuclei with many cerebral areas. We found that the functional connectivity decreased except for the sensory-motor cerebellum, which showed increased connectivity with some cortical areas. Among the three nuclei, only the decreased connectivity of the fastigial nucleus positively correlated with its decreased volume and negatively correlated with the axial symptom’s severity, indicating a relationship between the structural and functional alterations of the fastigial nucleus and the motor symptoms of PD already in the early stage of the disease. The supplementary analysis on the PPMI dataset confirmed the decreased cerebellar FC we found in the PD de novo patients.

The first novel finding of the present study is the atrophy of all three cerebellar nuclei. The finding of cerebellar atrophy was reported by previous studies performed in patients only with advanced PD^13,14^. In advanced PD patients, however, the finding is controversial and a meta-analysis of volumetric studies of the cerebellum in PD^15^ showed that the cerebellar volume loss is not a consistent feature of PD in their advanced stage. Differences in the types of analysis performed by the various authors may, however, account for the lack of consistent findings on cerebellar atrophy. For example, the segmentation study investigating single cerebellar subregion volumes, as we now did, has never been performed previously. Differently from previous voxel-based morphometric studies, we performed an ROI analysis using specific software dedicated to the analysis of the human cerebellum, which provides individual measures of cerebellar volumes based on a high-resolution atlas template of the cerebellum^16^.

The second novelty of the present study is the finding of DTI examination of cerebellar peduncles showing early white matter changes of the cerebellum’s major afferent and efferent pathways. The superior cerebellar peduncles consist mainly of efferent fibers directed to the cerebral cortex via the thalamus^17^ and to the contralateral red nucleus and inferior olive (dentato-rubro-olivary tract)^17^ whereas the middle and inferior cerebellar peduncles consist of afferent pathways from the spinal cord and cerebral cortex through the pons^17^. Abnormalities in the afferent and efferent cerebellar pathways may be critical in the pathophysiology of early PD.

By assessing cerebellum subregions’ functional connectivity, we observed that the sensorimotor and cognitive cerebellum displayed altered connectivity with areas involved in high cognitive function such as visual abilities (fusiform and lingual gyrus), emotion (cingulate cortex), motivation (accumbens), episodic and long-term memory (precuneus and thalamus) and action-monitoring processes (medial frontal cortex). The dentate nucleus also showed altered connectivity with the sensorimotor cingulate region (cingulate and paracingulate gyrus), parahippocampal gyrus and frontal medial cortex, i.e., associative areas which are normally implicated in cognitive functions^18,19^. Furthermore, we found that the fastigial and the interposed nuclei displayed altered connectivity with basal ganglia (putamen, pallidum and caudate) and other brain areas involved in motor control. Finally, fastigial and interposed nuclei showed decreased connectivity with cerebellar crus I and lobule VI which are mainly involved in executive functions such as working memory, planning and visuomotor functions^20,21^. Our findings suggest that distinct cerebellum subregions are involved in cognitive and motor network dysfunction in early PD.

By assessing possible correlations between structural and functional abnormalities, we found a positive correlation between fastigial nucleus atrophy and altered connectivity of the fastigial nucleus with right-VI and crus I. This finding suggests that changes in connectivity are related to volume loss, confirming previous evidence observed in advanced stages of PD^13^. Furthermore, there is also a negative correlation between the fastigial nucleus connectivity changes and postural disturbances. Indeed, the fastigial nucleus holds a key position in the axial, proximal and ocular motor control by projecting to the medial descending systems and eye movement related nuclei^22^.

Data on functional connectivity of cerebellum in PD are controversial. In keeping with our results some studies reported a decreased cerebellar FC^23,24^. On the other hand, other authors found an increased cerebellar FC^25–29^_,_ and others an increased FC with some regions and a decreased FC with others^30–32^. Moreover, some authors found a correlation between the increased FC connectivity and the clinical features of patients^26–29^ the presence or not of dopaminergic treatment^31,33^ while a decreased cerebellar FC has been related to a reduced cognitive performance^23^.

Taken together, studies on cerebellar connectivity in PD reveal that functional changes of FC connectivity of the cerebellum cannot be fully explained by the hypothesis of an initial compensatory connectivity followed by a decrease of FC as the disease progresses over time, as proposed by early task-related fMRI studies^34–36^. In fact, more recent resting state studies have reported both increased and decreased FC, sometimes in the same study, also depending on the considered brain areas^32^. In conclusion, the variability on FC findings in PD likely arises from differences in data analysis methodologies (e.g., seed-based, ICA, graph theory) and, from differences in the clinical features of patients studied^37^, making the results from the various studies difficult to compare. One strength of our study is that we have selected a homogeneous sample of patients all in the early stage of PD, therefore minimizing potential biases due to differences in disease duration or dopaminergic treatment. The supplementary analysis we performed in a different sample of PD de novo patients, obtained from the PPMI dataset, confirmed the decreased cerebellar FC in the early stage of the disease.

Regarding the MRI alterations previously described in early PD patients, several studies investigated other brain structures known to be involved in PD pathophysiology, such as basal ganglia and motor cortex^38–42^_._ Most studies did not find early structural basal ganglia abnormalities in the early stages^38,42,43^, whereas they revealed early functional alterations^40,44^. Similarly, functional rather than structural changes in the motor cortex were found^23^. Further studies simultaneously investigating the motor network structures, including the cerebellum, are needed to clarify the involvement of structural and functional MRI alterations in PD pathophysiology in the early stage.

The strengths of our study are the multimodal MRI approach on the cerebellar MRI alterations in early PD patients and the adequate sample size. However, our study has some possible methodological limitations. Firstly, because we used different MRI sequences for the evaluation of GM and WM alterations; therefore, future studies including multishell DTI sequence, which is ideal for a concomitant assessment of WM and GM microstructural changes, should be used to confirm these findings. Secondly, we have segmented two small nuclei such as fastigial and interposed nuclei using an automatic segmentation pipeline, thus we cannot ensure accuracy in these nuclei location as we did with the dentate nucleus.

Our study provided new evidence on the role of cerebellum structural and functional abnormalities in the early phase of PD, suggesting that different cerebellum regions contribute to the pathophysiology of axial and non-motor symptoms. These findings challenge the hypothesis that the cerebellum can have only a compensatory role in PD, at least at the early stage. Future longitudinal investigations are, however, needed to disentangle how the cerebellum intervenes in the pathophysiology of motor and non-motor symptoms during disease progression.

## Methods

Seventy-six patients with early PD and thirty-one age and sex-matched healthy subjects (HS) were enrolled. Descriptive statistics for demographic and clinical parameters in early PD and HS are reported in Table 1. Early-stage PD patients were identified on the basis of criteria already used in previous papers^45–47^. Inclusion criteria included: (i) age over 18 years, (ii) clinical history under two years, (iii) diagnosis according to international criteria^48^, and (iv) neurological examination by expert neurologists at a tertiary diagnostic center. Exclusion criteria were: (i) other neurological or psychiatric diagnoses, (ii) moderate to advanced PD (Hoehn and Yahr stage III-V), (iii) prior levodopa or other dopaminergic treatments, and (iv) presence of dyskinesia. Forty-five out of 76 patients underwent a SPECT CT DAT SCAN that confirmed PD diagnosis. None of the HS received treatment with drugs known to induce parkinsonism or were related to a case patient involved in the study.

A movement disorder specialist (SP or DB) performed the clinical assessment. The severity of motor symptoms was assessed by the MDS Unified Parkinson’s Disease rating scale (MDS-UPDRS) III, including bradykinesia, tremor, posture, and gait subscores. In particular, for bradykinesia we have summed the subitems of the UPDRS III from 3.4a to 3.8b, whereas for tremor we have summed from 3.15a to 3.18. For gait and posture we have used respectively the subitems 3.13 and 3.10. In contrast, non-motor symptoms were assessed by Non-Motor Symptoms Scale (NMSS). In addition, cognitive performance was assessed with the Montreal Cognitive Assessment (MoCA) and Frontal Assessment Battery (FAB).

Policlinico Umberto I Research Ethics Committee (Sapienza University of Rome) approved the study. All participants gave their written informed consent before participating in the study, which was conducted following the latest revision of the Declaration of Helsinki.

### MRI data acquisition

Images were acquired with a 3-Tesla (3 T) scanner (Siemens Magnetom Verio) and a 12-channel head coil designed for parallel imaging (GRAPPA). The following sequences were acquired:

- High-resolution three-dimensional (3D) T1-weighted (T1-3D) MPRAGE sequence (repetition time (TR) = 2,400 ms, echo time (TE) = 2.12 ms, inversion time (TI) = 1000 ms, flip angle = 8◦, field of view (FOV) = 256 mm, matrix = 256 × 256, 176 sagittal slices 1-mm thick, no gap);
- Diffusion tensor imaging (DTI) single-shot, echo-planar, the spin-echo sequence with ten interspersed volumes of b = 0 (b0) and 64 gradient directions, TR = 4,600 ms, TE = 78 ms, multiband acceleration factor = 2, monopolar diffusion scheme, FOV = 192 mm, matrix = 96 × 96, b = 1,000 s/mm^2, 72 contiguous axial 2-mm thick slices.
- Blood oxygen level-dependent (BOLD) single-shot echo-planar imaging (TR = 3,000 ms, TE = 30 ms, flip angle = 89°, FOV = 192 mm, 64 × 64 matrix, 140 volumes, voxel size = 3 mm^3^), with all patients instructed to close their eyes and remain awake during the resting-state functional MRI acquisitions.
- Dual turbo spin-echo, proton density (PD) and T2-weighted images (TR = 3320 ms, TE1 = 10 ms, TE2 = 103 ms, FOV = 220 mm, matrix = 384 × 384, 25 axial slices 4-mm thick, 30% gap).
- High-resolution 3D fluid-attenuated inversion recovery (FLAIR) sequence (TR = 6000 ms, TE = 395 ms, TI = 2100 ms, FOV = 256 mm, matrix = 256 × 256, 176 sagittal slices 1-mm thick, no gap).

An expert radiologist (PP) examined all MRIs, in particular T2-weighted and FLAIR images, to exclude the presence of concomitant brain lesions and focal white matter hyperintensities.

### MRI analysis

#### Data preprocessing

Structural and functional preprocessing was performed using *fMRIPrep* 20.2.3^49^, a toolbox based on *Nipype* 1.5.0; RRID: SCR_002502)^50^. Diffusion data were preprocessed using the FDT tools (FMRIB Diffusion Toolbox, part of FSL (FMRIB’s Software Library v.6.0.4, http://www.fmrib.ox.ac.uk/fsl/)^51^.

Structural preprocessing was performed using *fMRIPrep* 20.2.3; RRID:SCR_016216), which is based on *Nipype* 1.5.0 RRID:SCR_002502). Each of the T1-weighted (T1w) images were preprocessed with the following pipeline: first, the T1w image was corrected for intensity non-uniformity (INU) with N4BiasFieldCorrection, distributed with ANTs 2.2.0, RRID:SCR_004757), and used as T1w-reference throughout the workflow. The T1w-reference was then skull-stripped with a *Nipype* implementation of the antsBrainExtraction.sh workflow (from ANTs), using OASIS30ANTs as target template. Brain tissue segmentation of cerebrospinal fluid (CSF), white matter (WM) and gray-matter (GM) was performed on the brain-extracted T1w using fast (FSL 5.0.9, RRID:SCR_002823). Brain surfaces were reconstructed using recon-all (FreeSurfer 6.0.1, RRID:SCR_001847), and the brain mask estimated previously was refined with a custom variation of the method to reconcile ANTs-derived and FreeSurfer-derived segmentations of the cortical gray-matter of Mindboggle (RRID:SCR_002438). Volume-based spatial normalization to two standard spaces (MNI152NLin2009cAsym, MNI152NLin6Asym) was performed through nonlinear registration with antsRegistration (ANTs 2.2.0), using brain-extracted versions of both T1w reference and the T1w template. The following templates were selected for spatial normalization: *ICBM 152 Nonlinear Asymmetrical template version 2009c*, RRID:SCR_008796; TemplateFlow ID: MNI152NLin2009cAsym), *FSL’s MNI ICBM 152 non-linear 6th Generation Asymmetric Average Brain Stereotaxic Registration Model* (RRID:SCR_002823; TemplateFlow ID: MNI152NLin6Asym].

Functional preprocessing was also performed using *fMRIPrep* 20.2.3. For each subject, the following preprocessing was performed: first, a reference volume and its skull-stripped version were generated using a custom methodology of *fMRIPrep*. Head-motion parameters with respect to the BOLD reference (transformation matrices, and six corresponding rotation and translation parameters) are estimated before any spatiotemporal filtering using mcflirt (FSL 5.0.9). BOLD runs were slice-time corrected using 3dTshift from AFNI 20160207 (RRID:SCR_005927). The BOLD reference was then co-registered to the T1w reference using bbregister (FreeSurfer) which implements boundary-based registration. Co-registration was configured with six degrees of freedom. The BOLD time-series (including slice-timing correction when applied) were resampled onto their original, native space by applying the transforms to correct for head-motion. These resampled BOLD time-series will be referred to as preprocessed BOLD in original space, or just preprocessed BOLD. The BOLD time-series were resampled into standard space, generating a preprocessed BOLD run in MNI152NLin2009cAsym space. First, a reference volume and its skull-stripped version were generated using a custom methodology of fMRIPrep. Automatic removal of motion artifacts using independent component analysis (ICA-AROMA) was performed on the preprocessed BOLD on MNI space time-series after removal of non-steady state volumes and spatial smoothing with an isotropic, Gaussian kernel of 6 mm FWHM (full-width half-maximum). Corresponding “non-aggressively” denoised runs were produced after such smoothing. Functional preprocessed data were finally subjected to WM and CSF signal regression and high-pass filtering (100-seconds cut-off).

Diffusion data were visually inspected for artifacts and preprocessed using different tools from FDT (FMRIB Diffusion Toolbox, part of FSL (FMRIB’s Software Library v.6.0.4, http://www.fmrib.ox.ac.uk/fsl/). Images were corrected for eddy current distortion and head motion using a 12-parameter affine registration to the first no-diffusion weighted volume of each subject, and the gradient directions were rotated accordingly. Non-brain tissue was removed from the eddy-corrected images using the Brain Extraction Tool (BET), creating a binary mask of the brain. Head movement- and eddy-corrected images were fitted to the tensor model at each voxel to calculate fractional anisotropy (FA), mean diffusivity (MD), axial diffusivity (AD), and radial diffusivity (RD) maps using DTIFIT. Using the Tract-based spatial statistics (TBSS) pipeline, non-linear registration was performed to register FA images of all participants onto the standard-space image using FMRIB58_FA as the target image. First, affine-aligns the target image to 1x1x1mm MNI152 space. Once this is done, each subject’s FA image has the nonlinear transform to the target, and then the affine transform to MNI152 space is applied, resulting in a transformation of the original FA image into MNI152 space. Similarly, MD, AD, and RD maps were registered to the standard-space image to obtain similar images in the same standardized space. Lastly, a WM skeleton was generated from the mean FA image by thresholding at 0.2 to exclude GM or CSF.

### Cerebellar volumetric analysis

Cerebellar structures were identified, and volumes were calculated using the SUIT toolbox^16^. Cerebellar structures included 13 bilateral regions of the cerebellum (lobules I-IV, V, VI, VIIb, VIIIa, VIIIb, IX, X, Crus I, Crus II, and dentate, interposed and fastigial nuclei) and eight vermis regions. Each subject’s cerebellum was isolated and cropped from the 3DT1 anatomical images. Each cropped image was subsequently normalized into SUIT space using generated flow field and affine transformations. Lastly, the probabilistic cerebellar atlas was resliced back into the individual subject space to identify cerebellar lobules and nuclei. We carefully inspected SUIT outputs for each subject to ensure accuracy. The calculated cerebellar volumes were normalized to the total intracranial volume to reduce the effect of head size variability.

For further analysis, five regions of interest (ROIs) were chosen: sensorimotor cerebellum, cognitive cerebellum, fastigial, dentate, and interposed nuclei. According to the functional anatomy of the cerebellum^52,53^ the sensorimotor cerebellum was obtained by merging lobules I-V, VIIIa, VIIIb, while the cognitive cerebellum was obtained by merging lobules VI, VIIb, IX, X, Crus I, Crus II. Right and left fastigial, dentate, and interposed nuclei were merged to obtain a single ROI for each nucleus.

To ensure accuracy in dentate nucleus location, we performed a supplementary analysis by manually segmenting the dentate nucleus on T2* images. We applied a spatial cross-correlation analysis between the manually segmented and SUIT extracted dentate nuclei. The analysis showed a high spatial cross-correlation (r = 0.78), indicating a good correspondence between the dentate masks obtained with the two methods.

### Diffusion tensor Imaging of cerebellar peduncles

Using standard FDT and TBSS pipeline^54^ we calculated a white matter binary skeleton mask and 4D skeletonized images of fractional anisotropy (FA), medial diffusivity (MD), radial diffusivity (RD), and axial diffusivity (AD) (details are in supplementary material). Automatic tract-specific quantification using the John Hopkins University (JHU) WM tractography atlas was performed to identify the superior, middle and inferior cerebellar peduncles (SCP, MCP, ICP) and bilateral masks of these structures were created. Mean FA, MD, AD, and RD values were extracted for each subject using these masks.

### Functional connectivity analysis

Seed-based analyses were performed using FSL’s FMRI Expert Analysis Tool (FEAT). The mean time series for each subject was calculated within each selected 5 ROIs and used as seeds in the analyses. Voxel-wise maps of FC were calculated between each seed and the rest of the brain for each participant via a general linear model (GLM). The Harvard-Oxford Cortical and Subcortical Anatomical atlases included in FSL were used to determine the localization of significant clusters.

Furthermore, to compare our results with those collected by other authors we have carried out a FC analysis on a sample of 29 PD de novo patients and 20 HS using the Parkinson’s Progression Markers Initiative (PPMI) database (www.ppmi-info.org/data). We initially identified 30 PD de novo patients and 20 HS who underwent an MRI scan including both rs-fMRI and T1 weighted images before starting pharmacological treatment. One PD de novo patient was removed from the analysis because we found artifacts on the T1 weighted images.

### Statistical analyses

The Shapiro–Wilk normality test was performed to check for the normal demographic and clinical data distribution. Parametric and non-parametric tests were used for normally and non-normally distributed data. Sex differences were tested using the Chi-square test, while age differences were tested using the Mann–Whitney test. Differences between early PD and HS in cerebellar volumes as well as DTI measures (FA, MD, AD, and RD) were tested using the Mann-Whitney test. All results were false discovery rate (FDR) corrected.

Seed-based FC voxel-wise maps of the 5 ROIs were compared non-parametrically between patients and HS groups, using a two-sample unpaired t-test. Voxel-wise statistical analyses were performed via FSL Randomize permutation-based program with 5000 permutations, including age, sex and TIV as covariates of no interest. Results were corrected using FDR for multiple comparisons (*p* < 0.01). The minimum cluster extent was set at 100 voxels. The same statistical analyses were performed in patients obtained from PPMI dataset.

In our dataset, the Randomise-Tool (5000 permutations) was also used to examine the statistical correlations between significant FC alterations and structural MRI measures (cerebellar volumes in the 5 regions of interest and DTI measures of inferior, middle and superior cerebellar peduncles) as well as clinical scores (UPDRS III, bradykinesia, tremor, posture, gait, MoCA, NMSS scores. All correlation analyses were performed inside the masks of significant FC alterations. Regarding correlations between FC and structural MRI measures, FC maps of all 5 ROIs were correlated with their own normalized volume and FA scores of cerebellar peduncles. Furthermore, correlations between FC and clinical scores were performed as follows: (1) FC of the sensorimotor cerebellum was correlated with bradykinesia, tremor and UPDRS III score; (2) FC of the cognitive cerebellum was correlated with MoCA and NMSS scores; (3) FC of the fastigial nucleus was correlated with bradykinesia, gait, posture and NMSS; (4) FC of the interposed nucleus was correlated with tremor and UPDRS III; (5) FC of the dentate nucleus was correlated with bradykinesia, gait, posture and NMSS. The resulting statistical maps were corrected after FDR correction^55^ for multiple comparisons (*p* < 0.01).

The minimum cluster extent was set at 100 voxels. Finally, correlations between structural MRI measures (cerebellar volumes and FA, MD, AD, and MD of cerebellar peduncles) and clinical scores were performed using Spearman correlation with FDR correction (*p* < 0.01).

### Supplementary information

Supplemental material

## Data Availability

The datasets used during the current study available from the corresponding author are available on reasonable request due to privacy/ethical restrictions.
