# Supplementary material for: Understanding the role of cerebellum in early Parkinson’s disease: a structural and functional MRI study
Source: NPJ Parkinsons Dis. 2024 Jun 19;10:119. doi: 10.1038/s41531-024-00727-w (PMC11187155; doi:10.1038/s41531-024-00727-w)
Supplement: Supplementary file 1 — Supplemental material [file 41531_2024_727_MOESM1_ESM.pdf]

Supplementary Materials.

Supplementary Table 1. Cortical regions displaying higher connectivity of the sensory motor cerebellum in 76 de novo PD with respect to 31 healthy subjects (HS). ($p < 0.01$, FDR corrected, minimum cluster extent set at 100 voxels). Anatomical localizations of peak MNI coordinates were established according to Harvard–Oxford cortical and subcortical structural atlases and the cerebellar atlas included in FMRIB's Software Library.

**K**	**X**	**Y**	**Z**	**Brain Region**	**p**
186	20	-76	-10	Occipital Fusiform Gyrus(R)	<0.01
	22	-68	-4	Lingual Gyrus(R)	<0.01
103	-14	-64	-2	Lingual Gyrus(L)	<0.01

Supplementary Table 2. Cortical regions displaying lower connectivity ($p<0.01$, FDR corrected) of the sensory-motor cerebellum in 76 de novo PD patients with respect to 31 healthy subjects (HS). Refer to Supplementary Table 1 for a detailed explanation of the table layout.

**K**	**X**	**Y**	**Z**	**Brain Region**	**p**
110	62	-58	26	Angular Gyrus(R)	<0.01
301	-40	-56	18	Angular Gyrus(L)	<0.01
203	6	32	24	Cingulate Gyrus (R)	<0.01
	0	26	20	Cingulate Gyrus (L)	<0.01
	-2	52	24	Superior Frontal Gyrus(L)	<0.01
	-4	38	32	Paracingulate Gyrus(L)	<0.01

Supplementary Table 3. Cortical regions displaying lower connectivity (p<0.01, FDR corrected) of the cognitive cerebellum in 76 de novo PD patients with respect to 31 healthy subjects (HS). Refer to Supplementary Table 1 for a detailed explanation of the table layout.

**K**	**X**	**Y**	**Z**	**Brain Region**	**p**
370	-12	12	-8	Accumbens (L)	<0.01
	0	-8	2	Thalamus (L)	<0.01
259	4	-62	44	Precuneus Cortex(R)	<0.01
	0	-68	46	Precuneus Cortex (L)	<0.01
	8	-42	36	Cingulate Gyrus (R)	<0.01
989	4	48	-20	Frontal Medial Cortex (R)	<0.01
	20	66	-10	Frontal Pole (R)	<0.01
	-8	66	-10	Frontal Pole (L)	<0.01
	4	46	10	Paracingulate Gyrus (R)	<0.01
	-10	38	-10	Paracingulate Gyrus(L)	<0.01
387	-42	-10	-38	Inferior Temporal Gyrus(L)	<0.01
	-62	-10	-30	Middle Temporal Gyrus (L)	<0.01
117	-16	-102	-8	Occipital Pole(L)	<0.01

Supplementary Table 4. Cortical regions displaying lower connectivity (p<0.01, FDR corrected) of the dentate nuclei in 76 de novo PD patients with respect to 31 healthy subjects (HS). Refer to Supplementary Table 1 for a detailed explanation of the table layout.

K	X	Y	Z	Brain Region	p
126	-2	-22	-20	Midbrain (L)	<0.01
	-26	-8	-28	Hippocampus (L)	<0.01
	-24	-6	-22	Amygdala (L)	<0.01
	-30	-2	-18	Para-hippocampal Gyrus(L)	<0.01
	-36	-4	-10	Insular Cortex(L)	<0.01
	2	66	-14	Frontal Pole (R)	<0.01
705	4	46	-22	Frontal Medial Cortex(R)	<0.01
	-4	54	-16	Frontal Medial Cortex(L)	<0.01
	0	36	-12	Paracingulate Gyrus(R)	<0.01
	-10	36	-8	Paracingulate Gyrus(L)	<0.01
	6	38	-6	Cingulate Gyrus (R)	<0.01
	0	36	0	Cingulate Gyrus division(L)	<0.01

Supplementary Table 5. Cortical regions displaying lower connectivity ($p<0.01$, FDR corrected) of the interposed nuclei in 76 de novo PD patients with respect to 31 healthy subjects (HS). Refer to Supplementary Table 1 for a detailed explanation of the table layout.

K	X	Y	Z	Brain Region	p

	26	0	0	Putamen (R)	<0.01
579	-22	10	-2	Putamen (L)	<0.01
191	14	16	-4	Caudate (R)	<0.01
	20	2	-2	Pallidum (R)	<0.01
	48	-50	-32	Crus I (R)	<0.01
	36	-42	-38	Lobule VI (R)	<0.01
111	38	2	-16	Insular Cortex (R)	<0.01
	-40	6	-6	Insular Cortex(L)	<0.01
	-60	-22	22	Postcentral Gyrus(L)	<0.01
131	-10	32	-16	Frontal Medial Cortex (L)	<0.01
	-18	38	-16	Frontal Pole (L)	<0.01
215	8	-2	64	Supplementary Motor Cortex (R)	<0.01
	-2	-6	64	Supplementary Motor Cortex (L)	<0.01
	10	-4	72	Superior Frontal Gyrus(R)	<0.01
296	-48	24	26	Middle Frontal Gyrus (L)	<0.01
	-48	8	30	Precentral Gyrus(L)	<0.01

Supplementary Table 6. Cortical regions displaying lower connectivity (p<0.01, FDR corrected) of the fastigial nuclei in 76 de novo PD patients with respect to 31 healthy subjects (HS). Refer to Supplementary Table 1 for a detailed explanation of the table layout.

**K**	**X**	**Y**	**Z**	**Brain Region**	**p**

158	-12	-18	-20	Midbrain	<0.01
	-10	-6	-16	Amygdala (L)	<0.01
	-24	-8	-6	Pallidum (L)	<0.01
	-8	16	2	Caudate (L)	<0.01
249	26	-4	-2	Putamen (R)	<0.01
687	-22	4	-6	Putamen (L)	<0.01
280	44	-50	-32	Crus I (R)	<0.01
	30	-46	-30	Right VI (R)	<0.01
	42	14	-2	Insular Cortex (R)	<0.01
	-36	6	-2	Insular Cortex(L)	<0.01
	-42	20	-2	Frontal Operculum Cortex (L)	<0.01
	38	34	32	Middle Frontal Gyrus (R)	<0.01
	-54	18	32	Middle Frontal Gyrus (L)	<0.01
997	62	-24	32	Supramarginal Gyrus anterior division (R)	<0.01
	46	8	32	Precentral Gyrus (R)	<0.01
122	-4	-16	72	Precentral Gyrus (L)	<0.01
108	54	-32	32	Parietal Operculum Cortex (R)	<0.01
269	2	-70	48	Precuneus Cortex (R)	<0.01
	-12	-74	42	Precuneus Cortex (L)	<0.01
2016	6	0	32	Cingulate Gyrus (R)	<0.01
	-4	-8	32	Cingulate Gyrus (L)	<0.01

	10	-4	72	Superior Frontal Gyrus (R)	<0.01
	-22	6	56	Superior Frontal Gyrus (L)	<0.01
	2	-6	58	Supplementary Motor Cortex (R)	<0.01
	-10	6	56	Supplementary Motor Cortex (L)	<0.01

Supplementary Table 7. Significant positive correlation ($p<0.01$, FDR corrected) between fastigial FC and fastigial GM volume. Refer to Supplementary Table 1 for a detailed explanation of the table layout.

**K**	**X**	**Y**	**Z**	**Brain Region**	**p**
128	40	-50	-30	Crus I (R)	<0.01
	36	-42	-38	Lobule VI (R)	<0.01

Supplementary Table 8. Significant negative correlation ($p<0.01$, FDR corrected) between fastigial FC and Posture score. Refer to Supplementary Table 1 for a detailed explanation of the table layout.

**K**	**X**	**Y**	**Z**	**Brain Region**	**p**
103	48	-50	-32	Crus I (R)	<0.01
	30	-46	-30	Lobule VI (R)	<0.01

Supplementary table 9. Demographic and clinical characteristics of healthy subjects (HS) and early PD patients (PPMI dataset).

Demographic /clinical feature	HS (mean ± SD)	Early PD (mean ± SD)	p-value
**Age (years)**	61.01 ± 10.29	60.23 ± 9.62	0.26
**Gender (F/M)**	4/16	13/16	0.56
**UPDRS III**	-	18,42±10.38	-

UPDRS-III: Movement Disorder Society-sponsored revision of the Unified Parkinson's Disease Rating Scale, part III

Supplementary Table 10. Cortical regions displaying lower connectivity ($p<0.01$, FDR corrected) of the cognitive cerebellum in 29 de novo PD patients (PPMI dataset) with respect to 20 healthy subjects (HS). Refer to Supplementary Table 1 for a detailed explanation of the table layout.

K	X	Y	Z	Brain Region	p
140	60	-6	2	Superior temporal gyrus (R)	<0.01
	24	24	-14	Insular cortex (R)	<0.01
	12	-90	14	Occipital cortex (R)	<0.01

Supplementary Table 11. Cortical regions displaying lower connectivity (p<0.01, FDR corrected) of the dentate nucleus in 29 de novo PD patients (PPMI dataset) with respect to 20 healthy subjects (HS). Refer to Supplementary Table 1 for a detailed explanation of the table layout.

K	X	Y	Z	Brain Region	p
190	36	-38	-30	Lobule VI (R)	<0.01
142	36	-72	-48	Lobule VII b (R)	<0.01
117	40	10	-18	Insular cortex (R)	<0.01
100	-6	-96	2	Occipital cortex (L)	<0.01

Supplementary Table 12. Cortical regions displaying lower connectivity (p<0.01, FDR corrected) of the fastigial nucleus in 29 de novo PD patients (PPMI dataset) with respect to 20 healthy subjects (HS). Refer to Supplementary Table 1 for a detailed explanation of the table layout.

K	X	Y	Z	Brain Region	p
245	-4	-94	-2	Supracalcarin cortex (L)	<0.01
235	50	-56	12	Middle temporal gyrus	<0.01
211	42	54	-4	Frontal cortex (R)	<0.01
105	38	12	-18	Insular cortex (R)	<0.01

Supplementary Table 13. Cortical regions displaying lower connectivity (p<0.01, FDR corrected) of the interposed nucleus in 29 de novo PD patients (PPMI dataset) with respect to 20 healthy subjects (HS). Refer to Supplementary Table 1 for a detailed explanation of the table layout.

K	X	Y	Z	Brain Region	p
247	36	-86	8	Occipital cortex (R)	<0.01
124	-66	-32	-18	Middle temporal gyrus (L)	<0.01
120	38	58	-8	Frontal cortex (R)	<0.01
116	24	2	12	Putamen (R)	<0.01
